# Infections in Heart Failure - Impact on Mortality

**DOI:** 10.5935/abc.20180067

**Published:** 2018-04

**Authors:** Evandro Tinoco Mesquita

**Affiliations:** Universidade Federal Fluminense, Niterói, RJ - Brasil

**Keywords:** Heart Failure/mortality, Infections, Hospitalization, Sepsis, Shock,Septic, Infection Control

Infections represent an important emerging clinical problem that cause decompensation of heart failure (HF), and in many cases, life-threatening acute systemic disorder (sepsis) and septic shock. Cardiovascular system plays an important role in the development of multiorgan dysfunction in sepsis and refractory septic shock. Although intra-hospital death for sepsis decreased from 35% in 2000 to 18% in 2002, one third of patients die within one year after a septic event. Cardiovascular dysfunction significantly increases mortality rates in sepsis as compared with sepsis without cardiac dysfunction.^1^ Infection, *per se*, precipitates the occurrence of cardiac decompensation and is a direct marker of mortality in HF patients.^2^

The study by Cardoso et al.^3^ report a high hospital infection rate (45.8%) and relevant mortality (21,5%) among patients with decompensated HF. Interestingly, during the first year after hospital discharge, mortality rate was lower in patients with infection as compared with those without infection (11.5%*vs*. 22.2%; p = 0.04).

A recent study conducted with Murinae rodents showed myocardial injury, abnormal electrical conduction, cardiac dysfunction and increased cardiac apoptosis that may explain the cardiac instability observed in patients with severe infections. Studies have reported an interaction between the infectious agent, immune system and chemical mediators, with direct and indirect effects on myocardium.^1,4^

Clinical cardiologists have incorporated new criteria for early detection and treatment of sepsis and septic shock in HF, based on clinical protocols and imaging and microbiological tests, in addition to specific biomarkers. Elevated C protein (> 25 mg/mL) and procalcitonin levels are helpful in identifying infections as cause of HF decompensation.^5,6^

HF-related infections may be acquired in the community or during hospitalization, consisting primarily of pulmonary followed by urinary infections. From my clinical experience, infections on skin and intracardiac devices, and vascular access infections are relevant infection sites that should be examined in every patient with suspected infection.

In the present study, the authors show a group of inpatients with severe decompensated HF, who require high doses of inotropes, which makes the analysis of the real impact of sepsis/septic shock difficult. In the real world, many patients with severe HF (stage D) are at the end-of-life stage, where infections are common. For these patients, palliative care, instead of intensive care, is the most appropriate therapy. This reality is faced in our hospitals today and will certainly become more and more common in hospitals for chronic diseases in the future.

Some factors may explain the lower mortality seen in the post-hospital follow-up, including greater attention paid to these patients with infection (e.g. vaccination and healthcare services), and selection bias in which patients with more severe HF may have died during hospitalization due to infection. Arrigo et al.^7^ showing similar findings of lower mortality after hospital discharge involves a group of patients with decompensated HF associated with respiratory diseases (chronic obstructive pulmonary disease, asthma and pulmonary infection) rather than patients with respiratory infections only (without cardiac disease).

In summary, infections are an important cause of decompensation of HF that should be early detected and treated using specific protocols and in the presence of sepsis and/or septic shock. Volemic resuscitation, early antibiotic treatment, and referral to cardiac intensive care units are considered good clinical practices that can reduce the occurrence of hard outcomes such as death. On the other hand, it has become more and more recognized that sepsis promotes cardiovascular changes with multisystemic involvement that increase the frequency of cardiac and non-cardiac events after recovery from sepsis.
